# Exploration of Proton Pump Inhibitors Use During Pregnancy and Preeclampsia

**DOI:** 10.1001/jamanetworkopen.2021.24339

**Published:** 2021-09-07

**Authors:** Ahhyung Choi, Yunha Noh, So-Hee Park, Seung-Ah Choe, Ju-Young Shin

**Affiliations:** 1 School of Pharmacy, Sungkyunkwan University, Suwon, South Korea; 2 Department of Preventive Medicine, Korea University College of Medicine, Seoul, Republic of Korea; 3 Department of Clinical Research Design & Evaluation, Samsung Advanced Institute for Health Sciences & Technology, Sungkyunkwan University, Seoul, South Korea

## Abstract

This cohort study examines whether proton pump inhibitor use during pregnancy is associated with the rate of preeclampsia.

## Introduction

Preeclampsia affects 3% to 8% of pregnancies worldwide. Preclinical studies have suggested that proton pump inhibitors (PPIs) have preventive effects on preeclampsia^1^; however, studies in humans are limited and present conflicting results.^2,3,4^ Moreover, these studies have limitations, such as recall bias, residual confounding bias, and a lack of dosage information, which warrants additional studies to assess the association of preeclampsia and PPIs. Accordingly, we investigated whether PPI use during pregnancy is associated with the rate of preeclampsia.

## Methods

This cohort study was approved by the Sungkyunkwan University institutional review board. The need for informed consent was waived because this study was conducted using anonymized claims data. We followed the Strengthening the Reporting of Observational Studies in Epidemiology (STROBE) reporting guideline.

We conducted a nationwide cohort study using the Health Insurance Review and Assessment database of South Korea from 2011 to 2017, which covers the entire Korean population. We included all pregnant women aged 19 to 44 years who gave birth between 2013 and 2017. Exposure to PPIs was defined as at least 1 prescription during 4 windows: any time during pregnancy or during the first, second, or third trimesters of pregnancy. We considered 2 comparator groups: (1) women unexposed to PPIs from 90 days before the start of pregnancy to delivery, and (2) women exposed to an active comparator, histamine 2 receptor antagonist (H2RA). When comparing with H2RA-exposed group, women who received both PPI and H2RA during pregnancy were excluded from both comparator groups. To examine the dose-response relationship, we classified the exposure into 2 groups by cumulative defined daily dose (cDDD) of PPI (the ≤10 cDDD group and the >10 cDDD group).^5^ Study outcomes were all preeclampsia and preeclampsia accompanied with preterm birth, which were identified from 20 weeks of gestation. Propensity score (PS)-based fine stratification was used to adjust for 30 covariates, which are listed in the Figure.^6^ Relative risk (RR) with 95% CI was estimated using weighted generalized linear models. All analyses were conducted using SAS, version 9.4 (SAS institute).

**Figure.**
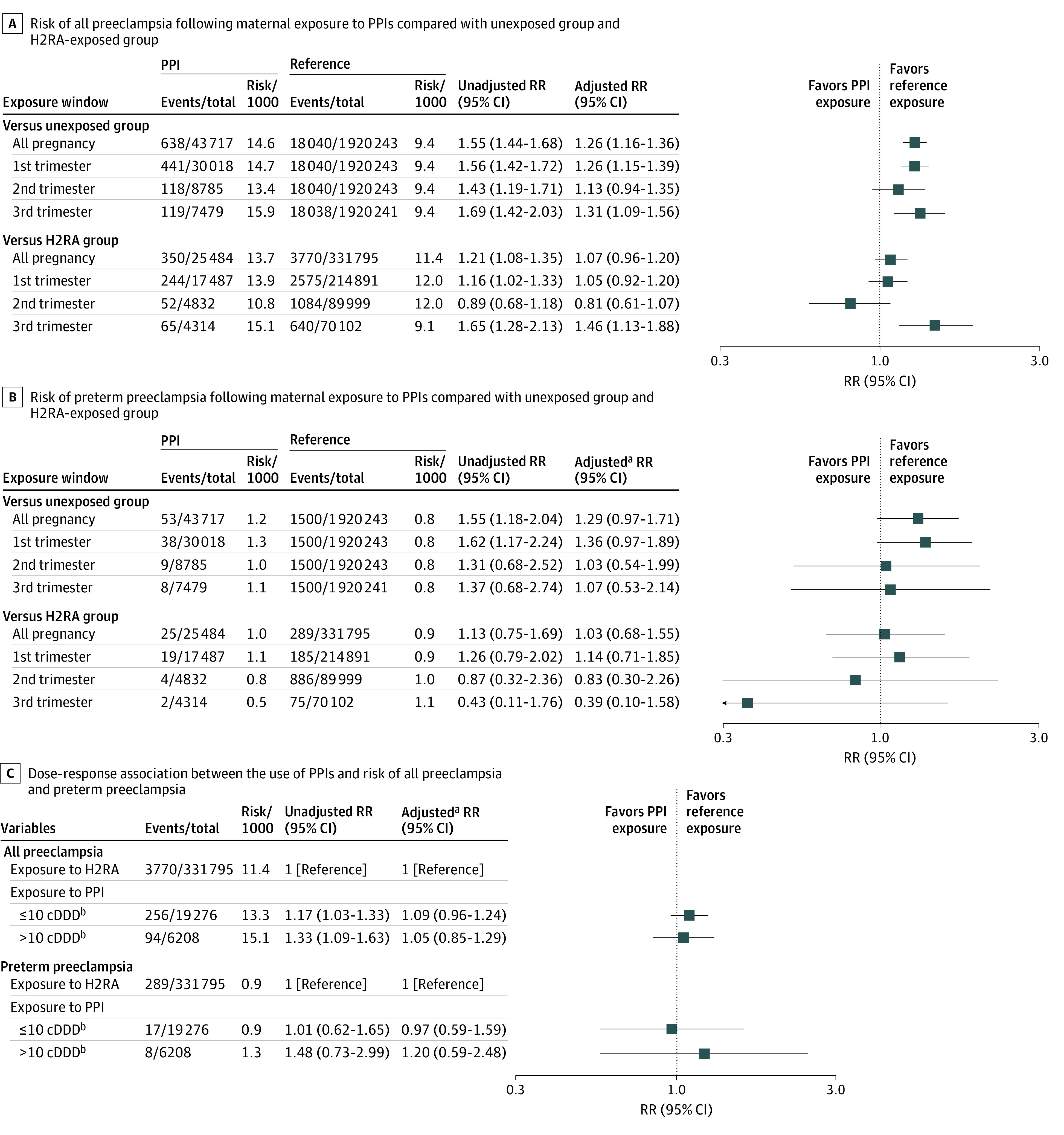
Risk of All Preeclampsia and Preterm Preeclampsia Following Maternal Exposure to PPIs and Dose-Response Relationship Between the Use of PPIs and Risk of All and Preterm Preeclampsia Abbreviations: cDDD, cumulative defined daily dose; H2RA, histamine 2 receptor antagonist; PPI, proton pump inhibitor; RR, relative risk. ^a^Adjusted for age and insurance type at delivery, nulliparity, multiple gestation, Charlson comorbidity index, indications for acid suppressive medications, including gastroesophageal reflux disease, heartburn, ulcer (eg, gastric, duodenal, peptic, and gastrojejunal ulcers and Zollinger-Ellison syndrome), maternal medical conditions (eg, asthma, anxiety, diabetes, depression, and chronic hypertension), inflammatory diseases (inflammatory bowel disease, systemic lupus erythematosus, rheumatoid arthritis), migraine/headache, renal disease, thyroid disorder, concurrent medications (eg, antidepressants, antiepileptics, antipsychotics, antihypertensives, antithrombotic agents, benzodiazepines, corticosteroids, oral hypoglycemics, insulin, nonsteroidal anti-inflammatory drugs, opioid analgesics, and disease-modifying antirheumatic drugs), and proxies of health care utilization (eg, the number of outpatient visits and hospitalizations). The proxies of health care utilization were assessed during a period of 180 days before the start of pregnancy, and other covariates were measured during the period of 180 days before the start of pregnancy to week 20 of the gestational period. ^b^cDDD is calculated as the sum of DDD prescribed during pregnancy.

## Results

Of 1 963 690 pregnancies, 43 717 (2.2%) were exposed to PPI during pregnancy (mean [SD] age, 31.7 [4.5] years). Women exposed to PPIs were more likely to have medical diagnoses (eg, gastroesophageal reflux disease) and to have used prescription drugs (eg, analgesics). After PS stratification, all baseline characteristics were well-balanced across all exposure contrasts with an absolute standardized mean difference of less than 0.1) In the PS-adjusted analyses, the RR for women exposed to PPI at any time during pregnancy was 1.26 (95% CI, 1.16-1.36) for all preeclampsia and 1.29 (95% CI, 0.97-1.71) for preterm preeclampsia, compared to non-users. However, when compared with H2RA-exposed group, the RR attenuated to 1.07 (95% CI, 0.96-1.20) and 1.03 (95% CI, 0.68-1.55) for all and preterm preeclampsia, respectively. For preterm preeclampsia, numerically decreased risk was observed in second trimester (0.83 [95% CI, 0.30-2.26]) and third trimester (0.39 [95% CI, 0.10-1.58]); however, the 95% CIs included the null value. For dose-response analysis, increased cDDD was not associated with a reduced risk of all preeclampsia or preterm preeclampsia compared with H2RA users.

## Discussion

In this nationwide cohort study, PPI use during pregnancy was not associated with a reduced risk of preeclampsia. We also did not find evidence of decreased risk with higher cumulative PPI exposure. However, as our study populations have taken the usual dose to manage gastrointestinal symptoms, further pharmacokinetic studies are required to determine the optimal dose for preeclampsia.

Our study has limitations. First, filled prescriptions do not necessarily mean actual use. However, unlike previous studies, PPIs are unavailable as over-the-counter drugs in South Korea, which may lead to relatively accurate exposure measurements. Second, although we adjusted for 30 covariates and used an active comparator, unmeasured confounding such as body mass index may be of concern. Third, estimates for preterm preeclampsia yielded wide 95% CIs because of the small number of preterm preeclampsia cases.

Overall, our findings suggest that PPI exposure during pregnancy is not associated with a lower risk of preeclampsia.
